# Impact of young adult life transitions on adult mental health problems: a propensity score analysis

**DOI:** 10.1017/S0033291725001072

**Published:** 2025-05-19

**Authors:** William E. Copeland, Shania Prytherch, W. Rothenberg, Jennifer W. Godwin, Lauren Gaydosh, Iliya Gutin, Guangyu Tong, Lilly Shanahan

**Affiliations:** 1 Department of Psychiatry, University of Vermont, Burlington, VT, USA; 2 Center for Child and Family Policy, Duke University, Durham, NC, USA; 3 Sociology Department, University of North Carolina, Chapel Hill, NC, USA; 4 Center of Policy Research, Syracuse University, Syracuse, NY, USA; 5 Cardiovascular Medicine, Yale University, New Haven, CT, USA; 6 Department of Psychology, University of Zurich, Zurich, Switzerland

**Keywords:** epidemiology, mental health, psychopathology, transitions, young adulthood

## Abstract

**Background:**

Mental health problems commonly persist from childhood to adulthood. This study tested whether young adult life transitions can improve adult mental health symptoms after adjusting for childhood mental health symptoms.

**Methods:**

The analysis uses data from the prospective, representative Great Smoky Mountains Study. Life transitions (e.g., high school completion, partnering, parenthood, and living independently) were assessed up to three times in young adulthood (ages 18 to 26; 3,241 observations). A cumulative variable counted the number of young adult transitions. Emotional, substance use, and antisocial personality symptoms were assessed at age 30 (1,154 participants or 81.2% of the original sample). Propensity models adjusted for early life adversities and psychiatric symptoms.

**Results:**

Multiple young adult transitions were common (m = 4.62; SD = 1.57). After adjusting for childhood mental health problems and adversities, each additional transition was significantly associated with a reduction in subsequent adult emotional symptoms (β = −0.34, 95% CI: −0.59, −0.08, *p* = 0.01) and adult antisocial personality disorder symptoms (β = −0.08, 95% CI: −0.14, −0.02, *p* < 0.001. These associations were stronger in males than in females. Young adult transitions were not associated with reductions in subsequent substance use symptoms (β = −0.04; 95% CI: −0.11, 0.03, *p* = 0.30). Young adult transitions related to educational milestones and consistent employment were associated with the largest reductions in symptoms.

**Conclusions:**

In this cohort study, life transitions during young adulthood were associated with reduced emotional and behavioral symptoms in adulthood. These transitions may constitute a potential mental health turning point and a specific, modifiable target for social policies.

## Introduction

Mental health problems constitute a significant public health burden in childhood, and these problems have the potential to affect both physical and mental health across the lifespan (Erskine et al., 2015; Harhay & King, 2012). Prospective longitudinal studies that have followed participants from childhood into adulthood consistently show, across cohorts and across the high-income countries, that common childhood psychiatric disorders are associated with an increased risk of adult disorders and poorer functional outcomes (Caspi et al., 2020; Copeland et al., 2013; Copeland, Shanahan, Costello, & Angold, 2009; Copeland, Wolke, Shanahan, & Costello, 2015). However, to date, there is little evidence that treatment of childhood mental health problems via services received in community settings improves adult mental health outcomes at all (Copeland, Tong, & Shanahan, 2022; Jörg et al., 2012). Therefore, the search continues for interventions, either through the community mental health system or naturally occurring, that might prospectively improve adult functioning. This study investigated whether achieving specific transitions or milestones during young adulthood has the potential to improve adult mental health functioning.

The period spanning from adolescence to adulthood is a pivotal juncture in an individual’s life, marked by profound changes and exploration (Arnett, 2000). Developmental psychologist, Jefferey Jensen Arnett, characterized the ages between 18 and 25 as “*emerging adulthood,”* distinct from both adolescence and young adulthood, as many individuals at this age report that they do not feel like an adult nor do they see themselves as adolescents (Arnett, 2000). During this period, some individuals experience increased autonomy, allowing them to make their own choices without the demands of adult responsibilities and giving them freedom to explore different experiences and pathways (Arnett, 2000). Others may face immediate financial and familial responsibilities. Still others may follow prescribed pathways focused on educational completion, employment, and partnering (Lee et al., 2023; Lee, Wickrama, O’Neal, & Prado, 2018). Despite these differences, young adulthood is a period when most young people navigate key educational, vocational, financial, and social transitions that often lead to adult roles and responsibilities. Mastering such transitions or milestones (e.g., college completion) has the potential to have a positive influence on mental well-being.

The study of young adult transitions has typically focused on individual transitions. For example, obtaining stable full-time employment (i.e., no periods of unemployment) has been associated with a decreased risk of substance use (i.e., cannabis, nicotine, and alcohol) and depressive disorders (Melchior et al., 2015; Howard, Galambos, & Krahn, 2010). Being married by age 32 is associated with higher life satisfaction in both men and women, whereas being divorced or separated is associated with reduced life satisfaction in women (Howard et al., 2010). Additional studies have highlighted the negative impact of failing to meet specific educational, vocational, and financial milestones (Cadigan, Duckworth, Parker, & Lee, 2019; Howard et al., 2010; Liem, Lustig, & Dillon, 2010; Melchior et al., 2015) or meeting them prematurely (e.g., parenthood (Grundström, Kiviruusu, Konttinen, & Berg, 2024). To date, only one study has examined the cumulative impact of multiple young adult transitions on adult functioning and found that an increased number of transitions was associated with a reduction in cannabis and illicit drug use at age 30 (Rothenberg et al., 2024). Such investigations are rare as they require studies which follow participants from childhood to adulthood while tracking mental health status and a broad range of educational, social, and employment-related transitions.

The current study leveraged a prospective cohort study that has followed participants through childhood/adolescence, young adulthood, and into adulthood to examine the impact of young adulthood life transitions on mental health and substance use symptoms after adjusting for child mental health and 24 early adversities. The study focuses on the impact of multiple transitions, as opposed to individual transitions one-at-a-time, to adjust for those who prioritize some transitions over others or those who lag on some transitions. Similar to Rothenberg et al. (2024), the study indexes a person’s cumulative rather than individual milestones. The study prospectively assessed – and adjusted for – childhood experiences, such as adversities and mental health problems that are associated with a person’s likelihood of navigating individual transitions. To stringently address our question, we employed the generalized propensity score (GPS) method that uses the inverse probability of treatment weights to balance young adults with varying numbers of positive transitions on the confounding characteristics (Imai & van Dyk, 2004). This approach approximates a randomized trial within an observational study and adjusts for nonrandomization.(Rosenbaum & Rubin, 1983; VanderWeele, 2006) As such, this study investigated the impact of cumulative life transitions net of the well-known impact of child mental health problems and adversities on both adult mental health and substance use problems.

## Methods

### Participants

This report follows the STROBE reporting guidelines for cohort studies (von Elm et al., 2008). The Great Smoky Mountains Study is a longitudinal, representative study of children in 11 predominantly rural counties of Southeastern US (see (Copeland, Angold, Shanahan, & Costello, 2014) that began in 1993. Three cohorts of children, ages 9, 11, and 13 years, were recruited from a pool of some 12,000 children using a two-stage sampling design, resulting in N = 1,420 participants (49% female participants; see also (Costello et al., 2003). First, potential participants were randomly selected from the population using a household equal probability design. Next, participants were screened for risk of psychopathology and participants screening high were oversampled in addition to a random sample of the rest. In addition, American Indians were oversampled to constitute 25% of the sample. Sampling weights were applied to adjust for differential probability of selection and allow results to generalize to the broader population (Copeland et al., 2014; Costello et al., 1996, 2003).

Annual assessments were completed with the participants, and their primary caregivers until participants turned 16 years old; thereafter only participants were interviewed at ages 19, 21, 25, and 30 (data from all are used here). An average of 83% of all possible interviews (N = 11,233) have been completed; by age 30, 39 participants had died. Additional information is provided in Supplementary Tables S1 and S2. Before interviews, all participants, and their parents (up to age 18) signed informed consent forms approved by the Duke University Medical Center ethical review boards. Participants received compensation for their time ($100 for most recent waves).

### Childhood/adolescent variables

The structured Child and Adolescent Psychiatric Assessment (CAPA) was completed with the participant and a parent in childhood/adolescence at annual assessments between ages 9 and 16 (Angold & Costello, 2000). The CAPA collected information on sex assigned at birth, race/ethnicity, parental educational attainment, parental income, overall family SES, and urban/rural status. The CAPA interview also assesses information related to child adversities and child psychiatric symptoms. In both cases, information was aggregated across all available observations to indicate if the child or parent ever reported the psychiatric symptom or the adverse experience.

**Childhood adversity** variables include scales indexing contextual risk factors (16 variables related to poverty, family structure, parental employment, out-of-home placement), family dysfunction (nine variables including poor parenting, parental conflict, neglect, and poor relationships between family members), and parent problems (nine variables including parent mental health, substance problems, and criminality).

The CAPA assesses **child psychiatric symptoms** over a 3-month primary period. A symptom was counted as present if reported by either parent or child or both. Scoring programs written in SAS combine information about the date of onset, duration, and intensity of each symptom to create DSM-IV diagnoses. A two-week test–retest reliability study of CAPA diagnoses in children aged 10 through 18 found kappa values ranged from 0.5 for conduct disorder to *Κ* = 1.0 for substance dependence (Angold & Costello, 1995). Common childhood psychiatric disorders assessed included anxiety disorders, mood disorders, conduct disorder, oppositional defiant disorder, ADHD, and substance disorders. For this analysis, child psychiatric symptoms were estimated with two variables: (1) **emotional symptoms** including all nonoverlapping symptoms of mood and anxiety disorders and (2) **behavioral symptoms** including all nonoverlapping symptoms of conduct disorder, oppositional defiant disorder, ADHD, and substance disorders. A similar set of emotional and behavioral symptom variables were derived based on the young adult observations.

### Young adult life transitions

Young adult transitions were assessed using the Young Adult Psychiatric Assessment (YAPA) (Angold et al., 1999), an upward extension of the CAPA interview administered to the participants at ages 19, 21, and 25. These transitions are related to educational, financial, vocational, social, and familial milestones that many (but not all) eventually meet. These milestones are often generally considered adaptive steps to adult roles and responsibilities. Table 1 lists the eight transitions, their descriptions, and their prevalence in the sample. The primary focus of this analysis was a cumulative life transition variable that summed the eight individual transitions.

**Table 1.** Description of young adult positive transitions

Transition	Description	N	%
Finish high school/GED	Completed high school or GED equivalent	1071	89.4
Complete some college	Completed at least some college-level coursework	676	63.9
Obtain steady employment	Has 30+ hours/week job with no history of being fired or quitting without another job	659	59.6
Live independently	Lives apart from home of origin	918	75.3
Attain financial stability	Reports good financial standing, paying bills on time and has health insurance	689	57.4
Have a steady partner	Has long term partner without significant substance use problems or criminality	337	28.9
Become a parent	Became a parent after age 20	514	33.9
Be connected socially	Reports frequent contact with peers, best friend and peer confidant	648	54.5

*Note:* N = 1281; Ns are unweighted and %s are weighted.

### Adult mental health symptoms

This study assessed psychiatric disorders and substance disorders at age 30. Adult variables were assessed using the YAPA. The assessment of adult psychiatric functioning resembled that of childhood disorders but relied only on self-reports. **Emotional symptoms** included any symptoms of major depressive disorder, dysthymic disorder, generalized anxiety disorder, panic disorder, social phobia, or specific phobia. **Substance symptoms** included DSM-defined substance use disorder symptoms related to nicotine, alcohol, cannabis, or an illicit drug (e.g. opioids or hallucinogens). To index externalizing symptoms, antisocial personality disorder symptoms (e.g., criminal behavior, lack of empathy, physical violence) were included.

### Analytic strategy

All statistical analyses accounted for the two-stage sampling design using sampling weights. Propensity score methods improve the covariate balance through matching, weighting, or stratification to make causal inferences in observational studies (Li, Morgan, & Zaslavsky, 2018; Rosenbaum & Rubin, 1983, 1984; VanderWeele, 2006). Here, we estimate causal effects for the ordinal exposure variable of cumulative transition using GPSs estimated via generalized boosted models (GBMs) (Imai & van Dyk, 2004; Zhu, Coffman, & Ghosh, 2015). The GPS estimation is achieved through the “twangContinuous” package (Coffman & Griffin, 2021), where the GBM is survey-weighted and set to have 10,000 trees, the amount of shrinkage as 0.01, and the interaction depth as 3 to allow for the exploration of sufficiently complex models. Covariates for the GBM include age, sex assigned at birth, race/ethnicity, parental educational attainment, parental income, overall family SES, urban/rural status, sum scores of childhood adversity variables (i.e., contextual risk, family dysfunction, parent problems), child psychiatric symptoms scores (i.e., total emotional and behavioral symptoms), and young adult psychiatric symptoms scores (i.e., total emotional and behavioral symptoms). The balance measure on covariates as a function of the number of iterations in GBM was checked to ensure satisfactory balance is achieved under our model setup (see Supplementary Figure S1). Balance of all covariates before and after applying for the GPS weights was assessed by Spearman’s correlation between each covariate and cumulative transition.

The GPS weights were then used to compute inverse probability weights. Inverse probability weights were combined with the survey weights for all outcome models (Austin, Jembere, & Chiu, 2018; Lenis, Nguyen, Dong, & Stuart, 2019), where all variables used to derive the propensity scores were included as covariates in the outcome models to ensure that results are doubly robust. Linear regression models were used for the symptom outcomes. Dose–response curves for each level of cumulative transition are presented for each outcome. In addition, each cumulative transition item was modeled independently as an exposure variable, and the propensity score estimation for each item was done through GBM with the same model specifications as that for the cumulative transition score. Balance checks on covariates based on standardized mean differences and survey-weighted linear outcome regression analysis were performed to estimate the effect of each single transition item. All analyses were performed on R 4.3.2 with adapted code from package “PSweight” for all outcome regression models (Zhou et al., 2020). Findings are considered statistically significant at *p* < 0.05, with point estimates and confidence intervals presented for all findings.

### Missing data

Of the original 1420 participants, all were assessed during childhood/adolescence (ages 9 to 16), 1281 (or 90.2%) were assessed in young adulthood (ages 18–27), and 1,154 (or 81.2%) were assessed in adulthood (at age 30). Participation in young adult or adult assessments was not associated with childhood mental health symptoms, sex assigned at birth, or race/ethnicity. Missingness of individual adult diagnostic or symptom items within completed adult interviews was rare (~1%). Twenty complete datasets were imputed to address missingness in both outcomes and covariates (Tong, Li, & Allen, 2022). R package “mice” was employed for both the imputation and synthesis of results in regression analysis (van Buuren & Groothuis-Oudshoorn, 2011).

## Results

### Patterns of young adult transitions

Table 1 displays the percentages of participants who had completed the different young adult life transitions between ages 19 and 25 (unweighted ns and weighted percentages). Many of the transitions were completed by more than half of the participants, including finishing high school, completing some college, obtaining steady employment, living independently, and being connected socially. Having a stable partner and becoming a parent were somewhat less common; about 30% of the participants reported each of these transitions. Overall, the mean number was 4.62 out of 8 possible positive transitions (SD = 1.57). A higher number of life transitions was associated with being female (4.98 versus 4.29, *p* < 0.01) and white (4.72 versus 3.84, *p* < 0.01). In zero-order correlations, cumulative young adult life transitions were significantly associated with lower subsequent levels of adult emotional and substance use symptoms (*r*s − 0.15, *p* < 0.01 and − 0.19, *p* < 0.01, respectively).

### Propensity score analysis

Table 2 shows the average Spearman’s correlations across the 20 imputed datasets between the covariates, as well as the cumulative transition variable before and after the application of the inverse probability of treatment weights obtained from the propensity score model. The unweighted correlations indicate strong associations (rho >0.5) between parental educational attainment and cumulative life transitions and small to moderate (*rs* 0.1 to 0.49) associations of the transition variable with symptom levels, sex, mean parental income, and other early adversity measures. This pattern suggests, as expected, that young adult life transitions are strongly associated with earlier family functioning, mental health functioning, and adversity exposure. All correlations were reduced to less than 0.1 after the application of the propensity score weights, suggesting desirable covariate balance (Table 2 and Supplementary Figure S2).

**Table 2.** Spearman weighted and unweighted correlations for covariates with and without inverse probability of treatment weights from propensity score modeling

	Overall sample		Males		Females	
Variable	Unweighted correlations	Weighted correlations	Unweighted correlations	Weighted correlations	Unweighted correlations	Weighted correlations
Sex	−0.216	−0.006				
Race	−0.078	−0.012	−0.086	−0.078	−0.040	−0.043
Ethnicity	−0.067	−0.014	−0.070	0.006	−0.085	−0.006
Cohort 2	−0.031	0.017	−0.057	0.011	0.016	−0.022
Cohort 3	0.033	0.005	0.029	0.060	0.024	−0.002
Parent educational attainment	0.509	0.093	0.512	0.072	0.460	0.065
Mean parental income	0.282	0.091	0.324	0.034	0.246	0.061
Overall SES	−0.248	−0.063	−0.293	0.010	−0.204	−0.023
Urban vs rural status	−0.008	−0.016	−0.044	−0.064	0.033	−0.018
Child adversities						
Contextual risk	−0.288	−0.070	−0.345	−0.024	−0.243	−0.027
Family dysfunction	−0.098	−0.014	−0.112	−0.024	−0.056	0.004
Parent problem	−0.200	−0.003	−0.265	−0.023	−0.131	−0.018
Psychiatric symptoms						
Child emotional sx.	−0.155	−0.048	−0.181	−0.053	−0.177	−0.014
Child behavioral sx.	−0.233	−0.044	−0.215	−0.083	−0.168	−0.022
YA emotional sx.	−0.222	−0.032	−0.211	−0.021	−0.143	−0.015
YA behavioral sx.	−0.076	−0.012	−0.097	−0.020	−0.149	−0.002

Abbreviations: YA = young adult. SES = socioeconomic status. sx = symptoms.

**Figure 1.**
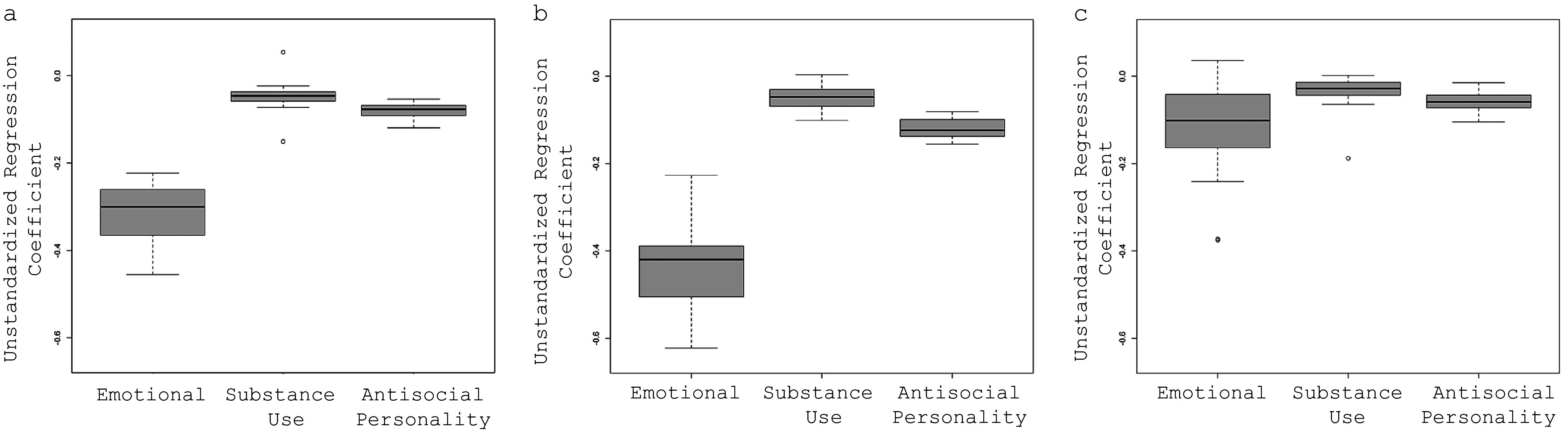
Average treatment effect of life transitions for emotional, antisocial personality, and substance-related psychiatric symptoms in adulthood for (a) the overall sample, (b) Males, (c) Females.

**Figure 2.**
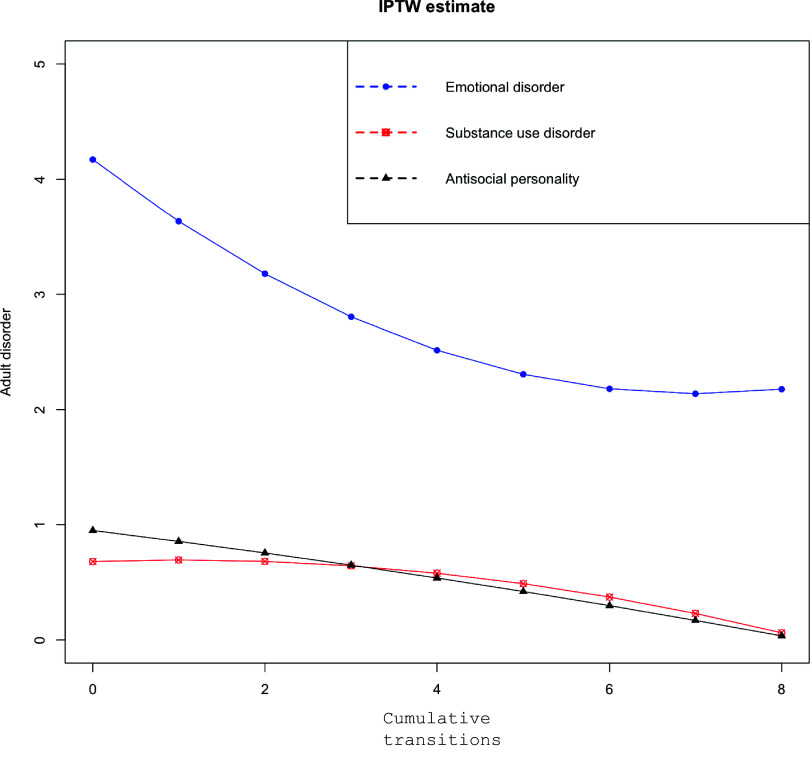
Dose–response relationship between number of life transitions and adult emotional, antisocial personality, and substance-use-related psychiatric symptoms.

Figure 1a–c shows the average treatment effect estimates of the cumulative young adult transition variable on adult emotional, substance use, and antisocial symptoms across the 20 imputed datasets. Values below zero indicate that the cumulative transition variable is associated with a *decrease* in adult psychiatric symptoms. The average treatment effect of the transition variable (pooled across the 20 imputed datasets using Rubin’s formula) on adult emotional symptoms was −0.34, 95% CI: −0.59, −0.08, *p* = 0.01. Thus, each additional life transition was associated with a reduction, by a third of a point, in the total emotional disorder symptoms. The average treatment effect of the cumulative life transition variable on adult substance use symptoms was not statistically significant (−0.04; 95% CI: −0.11, 0.03, *p* = 0.30). Finally, the average treatment effect of the life transition variable on adult antisocial personality disorder symptoms was −0.08, 95% CI: −0.14, −0.02, *p* < 0.001. The estimated average dose–response functions between cumulative young adult transitions and adult emotional, antisocial, or substance use symptoms are presented in Figure 2. The emotional symptoms decreased with each additional transition for up to 4+ life transitions, after which the association attenuated, while antisocial personality disorder symptoms continued to decrease linearly.

### Sex differences

Figure 1b,c shows the average regression coefficient estimates of cumulative young adult transitions on adult emotional and substance-related symptoms for *males* and *females.* The average treatment effects of the transition variable (pooled across 20 imputed datasets using Rubin’s formula) on male adults’ emotional symptoms was −0.43 (95% CI-0.76, −0.19, *p* = 0.01, which means that for each additional positive transition, there was nearly a half-point reduction in the total emotional disorder symptoms for males. However, the average treatment effect of the transition variable for females was of smaller magnitude and not statistically significant (−0.09; 95% CI-0.40, 0.21, *p* = 0.55. The average treatment effect of the cumulative life transitions on substance-related psychiatric symptoms was not significant for either males (0.04; 95% CI-0.14, 0.06 *p* = 0.42) or females (−0.03; 95% CI-0.14, 0.08 *p* = 0.62). The average treatment effect of the cumulative life transitions on antisocial personality disorder was significant for males (−0.12; 95% CI: −0.19, −0.04, *p* = 0.001) but not females (−0.06; 95% CI: −0.12, 0.01, *p* = 0.12). Additional models compared findings for different symptom groups in American Indian and non-American Indian groups (Supplementary Table S3). Significant associations for emotional and antisocial personality disorder symptoms were observed for the non-American Indian group only.

### Individual transitions

In the follow-up analyses, propensity score methods were applied to each of the individual life transitions to test which transitions had the strongest associations with adult symptoms. In terms of formal statistical significance, few individual young adult transitions showed significant effects on either emotional, antisocial personality, or substance-related symptoms in adulthood (see Table 3). In terms of effect size, individual life transitions with the largest average treatment effect sizes for emotional symptoms were completing high school, financial independence, and stable employment. For antisocial and substance use symptoms, the life transitions with the largest average treatment effects were completing some college coursework, completing high school, and stable employment. In contrast, the smallest average treatment effect sizes were associated with social transitions, including social connectedness, becoming a parent, and living independently.

**Table 3.** Associations of each individual positive transition with adult emotional, antisocial, and substance-related symptoms

	Emotional symptoms			Antisocial personality symptoms			Substance use symptoms		
Transition	B	Standard Error	95% CI	B	Standard Error	95% CI	B	Standard Error	95% CI
Social connectedness	−0.07	0.28	−0.62,0.49	−0.02	0.06	−0.13, 0.09	−0.13	0.18	−0.48,0.21
Stable job	−0.51	0.31	−1.12,0.11	−0.26	0.06	−0.37, −0.14	−0.23	0.19	−0.60,0.14
Healthy partner	−0.44	0.30	−1.03,0.15	−0.12	0.06	−0.23, −0.01	−0.19	0.13	−0.45,0.07
Financial independence	−0.65	0.35	−1.35,0.04	−0.12	0.06	−0.23, 0.00	0.06	0.17	−0.27,0.39
High school diploma	−0.93	0.78	−2.45,0.59	−0.30	0.16	−0.61, 0.01	−0.35	0.42	−1.17,0.47
Complete some college	−0.37	0.37	−1.09,0.35	−0.32	0.09	−0.50, −0.13	−0.93	0.78	−2.45,0.59
Becoming a parent	0.04	0.32	−0.59,0.67	0.02	0.07	−0.10, 0.15	−0.04	0.14	−0.31,0.23
Living independently	−0.09	0.35	−0.77,0.60	−0.07	0.06	−0.20, 0.05	−0.05	0.14	−0.33,0.23

## Discussion

There are good reasons to look at young adult life transitions as possible turning points for mental health symptoms (Laub & Sampson, 2003). The young adult period represents the handoff between development and maturity and between dependence and independence. It presents many individuals with the first opportunity to have more autonomy in making key life decisions that may support or undermine their mental health. This study tested whether transitions during this period could constitute turning points for improved mental health regardless of childhood struggles. Evidence was seen that young adult transitions may be turning points for some individuals: cumulative life transitions between ages 19 and 25 were associated with lower levels of subsequent emotional symptoms and antisocial personality disorder symptoms at age 30. This effect was generally limited to males and Non-American Indian participants and did not extend to substance use symptoms. These results provide a ray of hope that the choices and opportunities given to young adults can improve their subsequent health and well-being.

The current study advances our knowledge of young adult life transitions in the following ways. First, it prospectively followed participants throughout childhood and adolescence. This research design allowed us to account for individual factors, such as emotional and behavioral functioning in childhood and young adulthood and for family factors, such as family income and parental educational attainment, which were strongly associated with young adult transitions. Second, it assessed a range of young adult educational, financial, and social transitions throughout an extended period of young adulthood from ages 19 to 25. As such, this approach accounts for young adults who may prioritize some transitions over others and for young adults who may lag others in their developmental progression. Finally, this combination of prospective design, limited attrition, and in-depth assessment of childhood and young adulthood was coupled with the GPS method to provide a rigorous test of the impact of positive young adult transitions on subsequent mental health.

### Caveats

At the same time, these findings should be interpreted with caution for several reasons. First, this study included a representative sample of participants from 11 rural counties in North Carolina, but it is not representative of the U.S. population. African Americans and Latinos were underrepresented, and American Indians were overrepresented as compared to the US population. Second, the optimal design for testing the impact of positive adult transitions on mental health symptoms would involve a randomized trial. As that was not feasible, this study applied a causal analytic approach to propensity score modeling in the context of an observational longitudinal study. This approach approximates a randomized control trial to the degree that all relevant covariates can be balanced. While this study accounted for many childhood and young adult correlates of life transitions, unmeasured confounding is still possible. Finally, the valence of specific transitions may vary between individuals based on their life circumstances and cultural values. For example, the American Community Survey has consistently shown significant race and ethnicity differences in patterns of residential independence that are not accounted for by financial circumstances alone (Treas & Batalova, 2011). Similarly, the milestone of stable employment may run counter to the values of subpopulations where family responsibilities are prioritized over employment success. It is important to be careful and aware of the questionable assumptions about the valence of specific milestones.

The current findings affirm the importance of studying cumulative life transitions across multiple domains. The only other study that looked at multiple young adult transitions at the same times suggested a beneficial effect on adult cannabis and illicit drug misuse, but they did not look at emotional or antisocial personality symptoms (Rothenberg et al., 2024). In that study, the benefit of positive transitions was limited to the experience of 0–4 transitions with minimal additional benefit after 4+ transitions. This pattern is similar to that reported in other studies on the effects of protective factors earlier in the lifespan as well as associations of cumulative childhood risk factors/adversities with later health outcomes (Collishaw et al., 2007; Putnam, Harris, & Putnam, 2013). In each of these cases, the effect of individual risk or protective factors was found to be additive until a particular threshold of diminishing returns was reached. In this study, all the benefits of young adult transitions were limited to men. As noted above, the literature on positive young adults’ transitions is limited, especially on potential sex-specific effects. At the same time, young adulthood is a period of peak risk for males for substance use, criminality and other risky behaviors. Our findings suggest this period of peak risk may also be a period of second opportunities given the right social and educational, and employment contexts. Future studies of the impacts of positive transitions on mental health in adulthood should adjust for childhood mental health symptoms and test for sex differences to further understand which transitions have sex-specific effects and why.

Not all individual young adult transitions had similar effects on adult emotional and antisocial personality symptoms. The follow-up analyses suggested that the main drivers of improved symptoms were vocational, educational, and financial transitions. This conclusion was generally supported by previous studies of these individual milestones. Obtaining full-time employment has been found to protect against depressive disorders (Howard et al., 2010). Completing high school and obtaining a college degree have been found to be associated with a lower number of depressive symptoms than dropping out of high school or not going to college, even after controlling for disadvantages such as parental education level (Lee & Yang, 2022; Liem et al., 2010; McFarland & Wagner, 2015; Ou, 2008). On the other hand, social transitions such as having a strong support system and parenthood had minimal impact on mental health symptoms into adulthood. Research on these milestones has reflected their sometimes-heterogeneous impact. For example, parenthood has been linked to an increase in life satisfaction in the first year but to a decrease to below pre-pregnancy levels 3–4 years after (Clark, Diener, Georgellis, & Lucas, 2008; Dyrdal & Lucas, 2013). In addition, living independently by age 25 has not shown any benefit for later mental health (Grundström et al., 2024; Howard et al., 2010).

The origins of adult mental health problems often lie in childhood and adolescence but not always (Caspi et al., 2020; Copeland et al., 2013; Copeland, Shanahan, Costello, & Angold, 2009; Copeland, Wolke, Shanahan, & Costello, 2015). A key aim of population mental health research is to identify those interventions and experiences that have the potential to disrupt this common continuity of distress. Thus far, the candidates are limited, and the knowledge base is shallow. Even childhood mental health treatment has, thus far, failed to demonstrate such cross-developmental beneficial effects when studied in the real world (Copeland et al., 2022; Jörg et al., 2012). The current study suggests that some promising candidates may be naturally occurring and lying in the open. None of the transitions studied here are novel or necessarily out of reach for most young adults, but there are certainly more challenges for some than others. Most young adults – even those from challenging backgrounds – have already experienced multiple such life transitions. Moreover, these transitions appear to have a notable cumulative impact on psychiatric symptoms. Our findings suggest experiencing all six transitions could lead to an average of two symptoms of emotional disorders being reduced, regardless of one’s mental health history or the early adversity they faced. This current study suggests that efforts to support these transitions have the potential to pay dividends in reducing adult distress and promoting prosocial adult behavior. The aspirations of educational success and consistent employment are shared across the ideological spectrum providing a potential source of consensus and leverage for successful public policy efforts.

## Supporting information

Copeland et al. supplementary material Copeland et al. supplementary material

## Supplementary material

The supplementary material for this article can be found at http://doi.org/10.1017/S0033291725001072.
