# Supplementary material for: Impact of young adult life transitions on adult mental health problems: a propensity score analysis
Source: Psychol Med. 2025 May 19;55:e152. doi: 10.1017/S0033291725001072 (PMC12094619; doi:10.1017/S0033291725001072)
Supplement: Copeland et al. supplementary material [file S0033291725001072sup001.docx]

| Table S1. GSMS Participants followed up at each age | |
| --- | --- |
|  | N (%) |
| Original Sample | 1420 (100) |
| Age |  |
| 19 | 1072 (75.5%) |
| 21 | 1060 (74.6%) |
| 25 | 1109 (78.1%) |
| 30 | 1154 (81.2%) |

| Table S2. Basic information about the Great Smoky Mountains Study | |
| --- | --- |
|  |  |
| Total # of participants | 1,420 |
| Sampling strategy | Community, representative with oversampling for children at risk for mental health problems |
| Design | Accelerated longitudinal with 3 cohorts |
| Observations | 11,233 (Median=8 per participant; Interquartile range 7-10) |
| Informant | Ages 9-16: Parent and Self-Report;  Ages 19-25: Self-report only  Age 30: Self-Report only |
| Age | Cohorts aged 9 (N=508), 11 (n=497), and 13 (N=415) at baseline; Average age at last adult assessment: 30.9 (SD=1.7; range 26-35) |
| Sex | 49.2% female |
| Race and Ethnicity | 89.8% White; 6.4% African-American; 3.8% American Indian |
| Interview | Ages 9-16: Child and Adolescent Psychiatric Assessment  Ages 19-25: Young Adult Psychiatric Assessment  Age 30: Young Adult Psychiatric Assessment |

| Table S3: Effect size of positive transition in adulthood by race/ethnicity | | | | | | |
| --- | --- | --- | --- | --- | --- | --- |
|  | American Indian Participants | | | Non-American Indian participants | | |
|  | Effect Size | Standard Error | 95% CI | Effect Size | Standard Error | 95% CI |
| **Symptoms** |  |  |  |  |  |  |
| Emotional | -0.01 | 0.10 | -0.22, 0.20 | -0.33 | 0.15 | -0.63, -0.03 |
| Substance-related | 0.01 | 0.06 | -0.11, 0.14 | -0.05 | 0.06 | -0.17, 0.07 |
| ASPD | -0.06 | 0.04 | -0.13, 0.01 | -0.08 | 0.03 | -0.14, -0.02 |


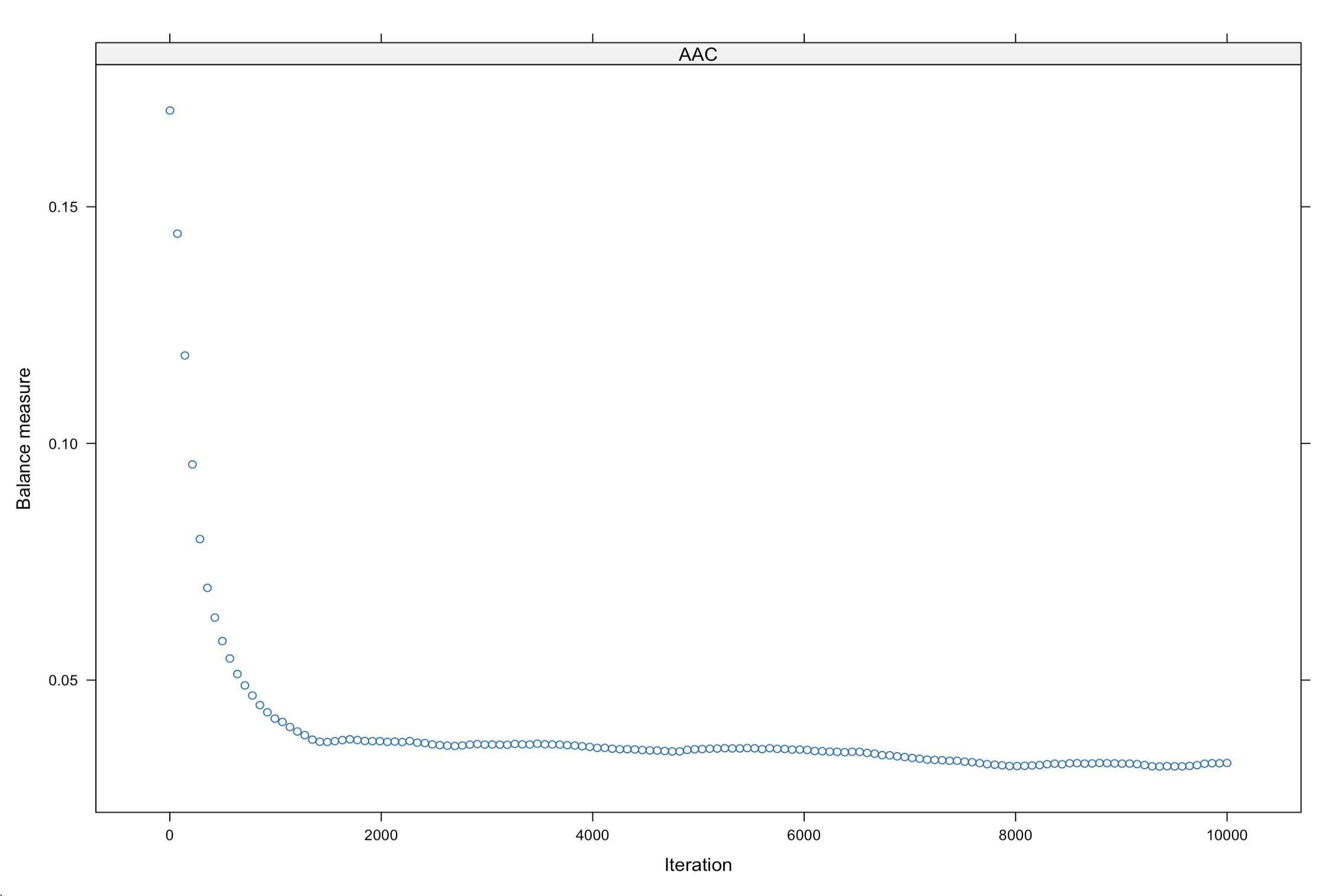


**Figure S1.** The average balance of covariates in absolute Spearman’s correlations is over 10,000 iterations of the generalized propensity score model using the generalized boosting method.


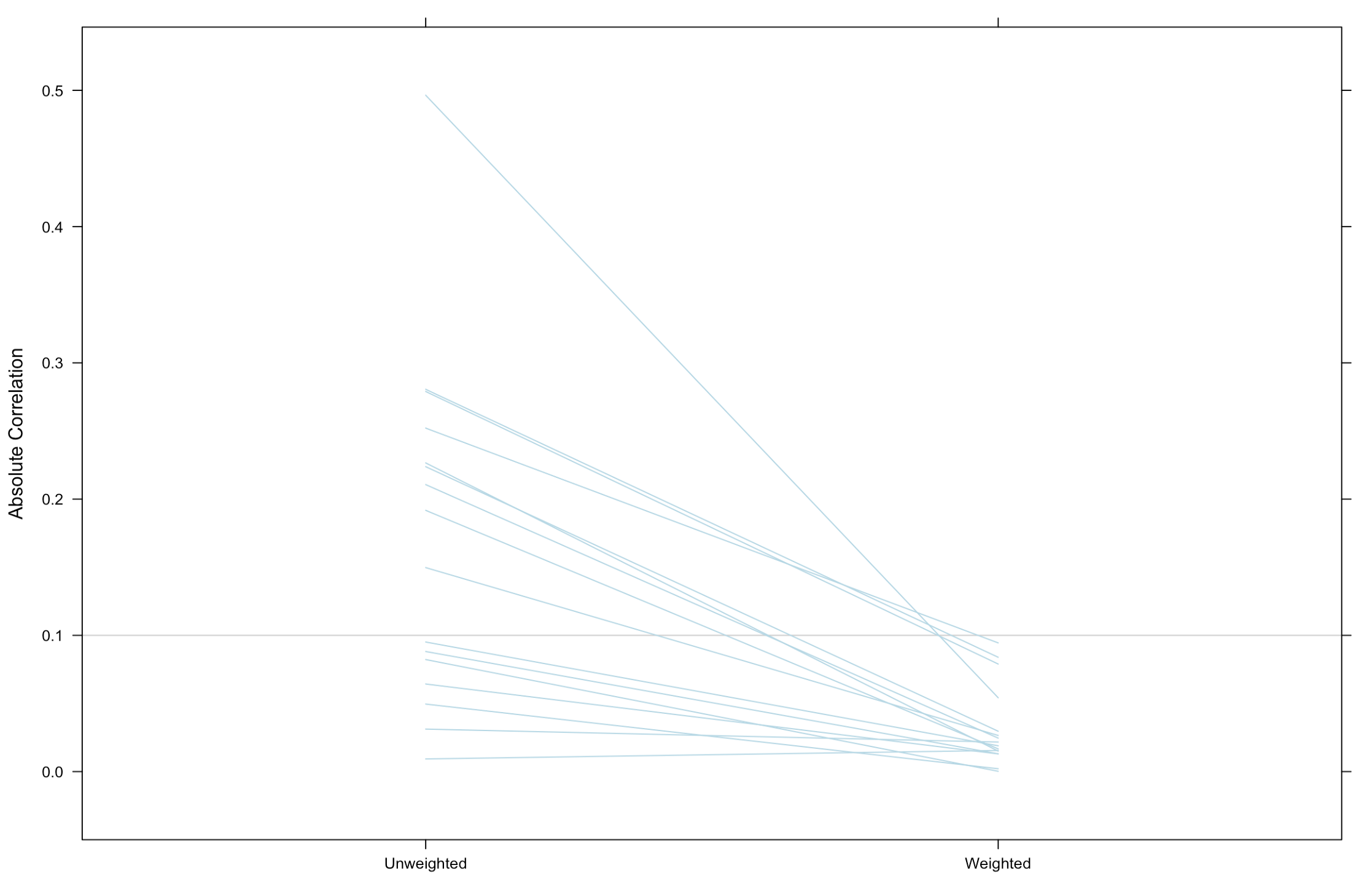


**Figure S2.** Absolute Spearman’s correlations of covariates with the transition before and after applying the weights obtained from the propensity score model using the generalized boosting method.
